# Nevus Depigmentosus Treated by Melanocyte–Keratinocyte Transplantation

**DOI:** 10.4103/0974-2077.79185

**Published:** 2011

**Authors:** Sanjeev V Mulekar, Ahmed Al Issa, Abdullah Al Eisa

**Affiliations:** *National Center for Vitiligo and Psoriasis, Olaya District, Tahlia Street, Riyadh, Kingdom of Saudi Arabia*

**Keywords:** Grafting, melanocyte transplantation, nevus depigmentosus, nevus treatment, nevus

## Abstract

**Background:**

Nevus depigmentosus is a congenital, nonprogressive hypopigmented disorder. Various therapeutic methods have been attempted to repigment nevus with variable results.

**Objective:**

The objective of this study is to report our experience of treatment of nevus depigmentosus with a combination of noncultured melanocyte–keratinocyte transplantation (MKTP) and excimer laser sessions.

**Materials and Methods:**

Six patients (male 1, female 5) of nevus depigmentosus were treated with a combination of noncultured melanocyte–keratinocyte transplantation and excimer laser. One patient was lost to follow-up. Remaining five patients were observed for a period ranging from 7 to 30 months.

**Results:**

Two patients responded poorly to MKTP. The remaining three patients responded with repigmentation ranging from 80% to 100% but the quality of repigmentation was unsatisfactory in two of them.

**Conclusion:**

Though repigmentation of nevus depigmentosus is possible by grafting techniques, the results are inconsistent and recurrence is possible.

## INTRODUCTION

Nevus depigmentosus is a congenital, nonprogressive cutaneous hypomelanosis, which can present as a circumscribed irregular, oval, or round or as a unilateral band or streak with a block-like configuration or arranged along Blaschko’s lines.[1] It affects males and females equally with no known patterns of inheritance or familial tendency. The lesions are hypomelanotic and remain unchanged throughout life although they may enlarge proportionately in a growing child. Wood’s lamp examination of nevus depigmentosus shows off-white accentuation, in contrast to the chalk-white accentuation observed in vitiligo.

Therapeutic attempts to repigment nevus depigmentosus have been made with PUVA, excimer laser, and surgical grafting methods. We report our experience of nevus depigmentosus treated with a combination of noncultured melanocyte–keratinocyte transplantation (MKTP) and excimer laser with the review of the literature.

## MATERIAL AND METHODS

Six patients (male 1, female 5) of nevus depigmentosus were treated between July 2006 and March 2008 with a combination of MKTP and excimer laser. The ethics committee of the National Centre for Vitiligo and Psoriasis (NCVP) approved the study and all patients signed an informed consent. Informed consent was obtained from parents of patients younger than 18 years of age. All these patients were clinically diagnosed based on the criteria proposed by Coupe in 1976[2] [Figures 1a and 2a]. Following information was recorded for each patient: age, sex, age at onset, anatomical location, and any change in the size and shape of the lesion [Table 1]. One patient was lost to follow-up. Remaining five patients were observed for a period ranging from 7 to 30 months. These patients were treated with excimer laser sessions administered three times weekly starting 3 months postoperatively for a period of 6 weeks. To improve upon the quality of pigmentation 1 year after the first procedure, repeat MKTP was performed for patients 1 and 3.The lesions were photographed before MKTP and at the end of the respective follow-up visit and were assessed by two authors (SM and AI) for the percentage of repigmentation.

**Table 1:** Demographic data of patients

No./age/sex	Anatomical site	Age at onset	Size and shape
1/13/F	Left cheek, forehead, nose	Since birth	Unchanged
2/21/F	Right cheek	Since birth	Unchanged
3/3/F	Right shoulder, upper chest	Since birth	Unchanged
4/6/F	Left side of the neck, jaw line	Since birth	Unchanged
5/5/M	Shoulder to nape	Since birth	Unchanged
6/12/F	Right leg	Since birth	Unchanged

**Figure 1a:**
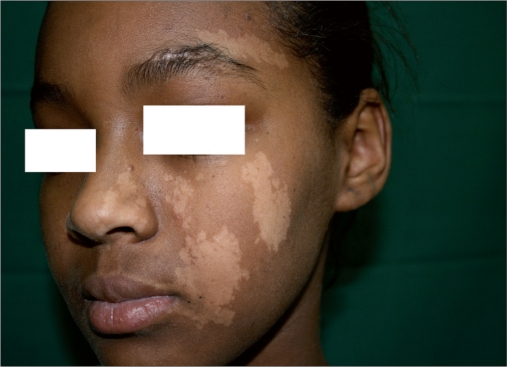
13-year-old African female child before treatment

**Figure 2a:**
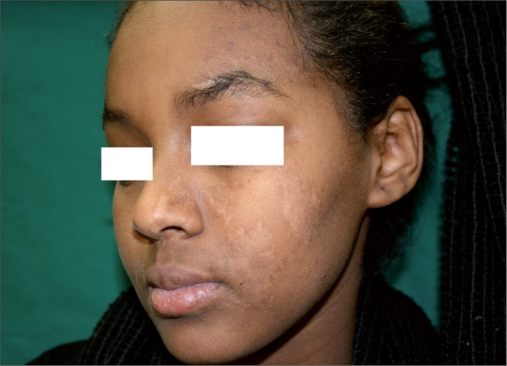
3-year-old Saudi female child before treatment

## SURGICAL TECHNIQUE

The whole procedure, which consists of harvesting the donor skin sample, preparation of cell suspension, and recipient area, and the transplantation of the cell suspension, was performed in a clean procedure room. No special laboratory set-up was used for the cell separation procedure.

### Harvesting the donor skin sample

A donor skin one-tenth of the recipient area was marked on the lateral aspect of the gluteal region, and was anaesthetized with 1% lidocaine injection. The skin was stretched, and a very superficial skin sample was taken with Silver’s skin grafting knife (E. Murray & Co., Cork, UK). The superficial wound was then covered with Duo-DERM (Convatec, Bristol-Myers Squibb Co., USA) extra thin dressing followed by sterile gauze pieces and micropore dressing (3M, USA).

### Preparation of the cell suspension

The thin skin sample was transferred to a Petri dish containing approximately 5 ml of the 0.25% (weight/volume) trypsin solution. The sample was incubated for 25 min at 37°C in an incubator. After incubation, the Petri dish was taken out, and trypsin removed with a Pasteur pipette. The skin sample was then washed with Dulbecco’s modified Eagle’s medium F12 (DMEM/F12) (Invitrogen Corp., Carlsbad, CA, USA) to remove traces of trypsin. The dermis was separated from the epidermis; the dermis was then discarded and the epidermis was broken into multiple pieces with tweezers. The epidermal pieces were then transferred to a 15-ml centrifuge tube (Tarson Products Pvt. Ltd, Kolkata, India) and centrifuged for 5 min. The epidermal cells settled at the bottom of the centrifuge tube in the form of a pellet, which was then suspended in DMEM/F12 in a 1-ml syringe with a detachable needle. The size of the recipient area determined the quantity of the suspension prepared.

### Preparation of the recipient area

The recipient site was cleaned with povidone iodine and 70% ethanol and anaesthetized with 2% lidocaine injection. It was then dermabraded superficially with a high-speed dermabrador fitted with a diamond fraise wheel to the level indicated by pinpoint bleeding spots. The denuded area was covered with sterile gauze pieces moistened with the isotonic sodium chloride solution until the transplantation of the cell suspension.

### Transplantation of the cell suspension

The cell suspension was applied to the dermabraded recipient area and covered with a dry collagen sheet (Neu skin^®^ F; Eucare Pharmaceuticals, Chennai, Tamil Nadu, India). A biodegradable collagen sheet assists the attachment of transplanted cells to provide an optimal environment for cellular growth and vascularization. A secondary dressing of sterile gauze pieces secured with a micropore adhesive tape was applied. Dressing was removed after 1 week. Absolute immobilization was not necessary.

## RESULTS

The results are described in Table 2. Patients were assessed for the repigmentation 3 months after the MKTP. All the patients were treated with excimer laser because the lesions appeared substantially hypopigmented to the naked eye, though Wood’s lamp examination showed significant pigmentation. Patients 5 and 6 responded poorly to MKTP. The remaining three patients responded with repigmentation ranging from 80% to 100% [Figure 1b and 2b]. Patient 1 experienced loss of pigmentation 2 months after the discontinuation of phototherapy [Figure 1c]. Therefore, she was treated again with MKTP, which was followed by excimer laser sessions. The quality of repigmentation was unsatisfactory in two of three patients.

**Figure 1b:**
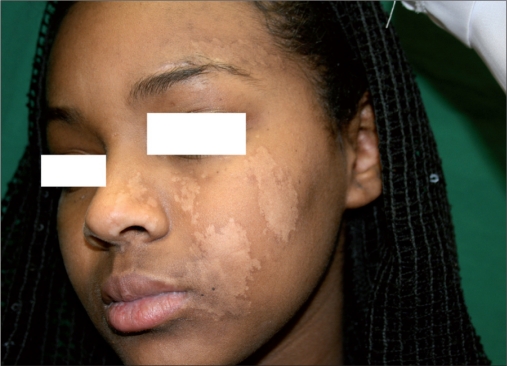
3 months posttransplantation and postexcimer laser. Note good repigmentation

**Figure 2b:**
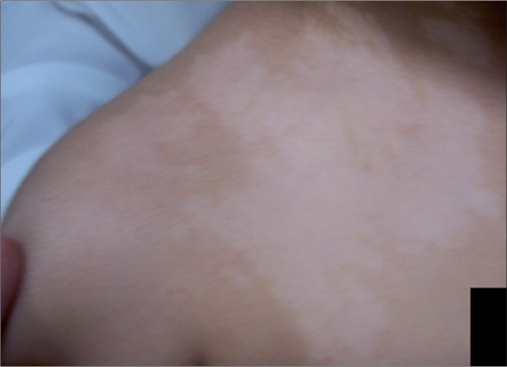
6 months posttransplantation with good repigmentation

**Figure 1c:**
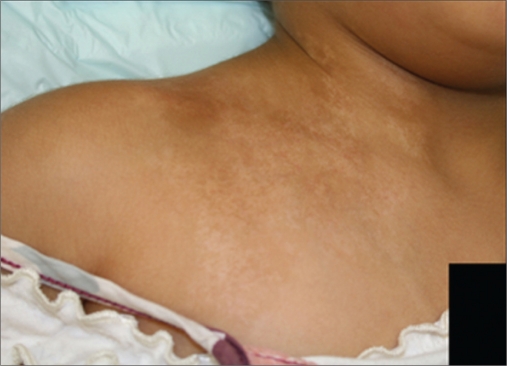
7 months posttransplantation. Note partial loss of pigmentation

**Table 2:** Results of patients treated with MKTP

No./age/sex	Area treated (cm^2^)	Repig.%	Recurrence	Quality of pigmentation	Follow-up period (months)	Post-MKTPexcimer laser
1/13/F	30.75	100	Yes	Colour mismatch	7	Yes
2/21/F	13.50				No	
3/3/F	35.0	100	No	Good	30	Yes
4/6/F	15.0	80	No	Colour mismatch	11	Yes
5/5/M	89.0		No	Poor	8	Yes
6/12/F	12.0		No	Poor	25	

Repig.: repigmentation; MKTP = melanocyte–keratinocyte transplantation

## DISCUSSION

The exact cause of nevus depigmentosus is still not clearly understood. A sporadic defect in the embryonic development has been suggested to be a causative factor.[3] Most investigators agree that there is no change in the number of melanocytes[4,5] in the affected area, and report abnormal melanosome transfer resulting in their decreased number in keratinocytes[4] or reduction in the number of melanosomes in melanocytes.[5] However, Takaiwa and Mishima reported decreased number of melanocytes without any disturbance in the melanocyte transfer to keratinocytes.[6] In a similar observation by Sung *et al*., melanocyte counts were significantly decreased when stained with NKI/beteb and MART-1. It was mildly decreased when stained with the S-100 protein stain.[7]

Different therapeutic modalities have been attempted to repigment the lesions of nevus depigmentosus such as PUVA, excimer laser, and different grafting techniques. PUVA therapy has not been shown to be beneficial.[8] Successful repigmentation was reported in a single case with 14 sessions of excimer laser treatment.[9] Repigmentation of nevus with blister roof grafting[10,11] and cultured epidermal grafting[12] has been reported, but the quality of repigmentation was not satisfactory and one of the treated patients experienced loss of repigmentation in the following years.[10] Though Gauthier reported successful repigmentation of nevus depigmentosus treated by noncultured MKTP,[13] Olsson and Juhlin[14] reported failure with the similar technique. Falabella has observed that nevus depigmentosus does not respond to melanocyte grafting.[15]

In this series, we observed inconsistent results with three patients showing the pigment spread ranging from 80% to 100% while two patients responded poorly. However, the quality of pigmentation was not satisfactory in two of three responders. All the patients were treated with excimer laser postoperatively because the resultant pigmentation was lighter compared to the surrounding skin color. The quality of pigmentation improved in all these patients after excimer laser treatment. One patient experienced partial loss of pigment after the discontinuation of laser sessions. She responded with good repigmentation after she was treated again with MKTP and excimer laser sessions.

Previous reports of various grafting methods also show results which vary from a good pigment spread with an unsatisfactory quality of repigmentation to failure to repigment and recurrence in the following years. The data of therapeutic attempts with phototherapy are scanty. The attempt to repigment nevus with PUVA has failed and there is only a single case report with successful repigmentation of nevus depigmentosus treated with excimer laser.

The transplantation or grafting is performed with the hypothesis that the melanocytes from the uninvolved skin are not affected by the unknown factor responsible for the abnormal transfer of melanosomes and continue to function normally at the transplanted site. However, in practice we observe variable results with poor quality pigmentation. Thus, further research is required to understand the pathogenesis of development of the hypopigmented nevus.

Though the repigmentation of nevus depigmentosus is possible by grafting techniques, the results are inconsistent and recurrence is possible. In consideration of the experience of other authors and us, the quality and retention of pigment are unpredictable. These factors need to be considered while consulting and offering any treatment to the patient of nevus depigmentosus.
